# The Role of Social Networks in Facilitating Personal Trust

**DOI:** 10.1093/geroni/igaf122.1862

**Published:** 2025-12-31

**Authors:** Noah Webster, Kristine Ajrouch, Toni Antonucci

**Affiliations:** University of Michigan, Ann Arbor, Michigan, United States; University of Michigan, Ann Arbor, Michigan, United States; University of Michigan, Ann Arbor, Michigan, United States

## Abstract

Trust is a core component of social cohesion and a strong predictor of older adults’ well-being. Most research on trust focuses on others who are geographically proximate, with less focus on those in one’s country more generally, i.e., macro-level personal trust. Larger-scale trust is critical for maintaining social norms, ensuring effective institutions, and civic engagement. In this study, we integrate two perspectives on social capital by examining the association between social network structure (e.g., size, composition) and trust of ‘people in the U.S.’ and the extent that trust clusters within close social networks. Data are from adults age 60 and older from Wave 3 of the Detroit-based Social Relations Study collected in 2015 (n = 303). Respondents identified social network members based on varying levels of closeness and provided characteristics on up to the first 10 people mentioned. Additionally, up to three network members age 18 plus were interviewed and also asked about personal trust. Networks with at least one person age 60 and older were selected for analysis (n = 162 networks; 425 respondents). Regression analyses controlling for age, gender, marital status, race, and education indicate having a larger inner circle size (i.e., more people one cannot imagine life without) and a greater proportion of friends in one’s network were significantly associated with greater trust. Further, examination of a random intercept model predicting trust indicated significant clustering of trust within close social networks (intra class correlation = .21). Findings suggest fostering close non-family ties can be an effective approach to building societal-wide trust.

